# Biopolymeric Mucin and Synthetic Polymer Analogs: Their Structure, Function and Role in Biomedical Applications

**DOI:** 10.3390/polym8030071

**Published:** 2016-03-02

**Authors:** Sundar P. Authimoolam, Thomas D. Dziubla

**Affiliations:** Department of Chemical and Materials Engineering, College of Engineering, University of Kentucky, 177. Paul Anderson Tower, Lexington, KY 40506, USA; sundar.prasanth@uky.edu

**Keywords:** mucin, biomimic, bioapplication, polymer analog, polymer networks, tissue engineering, drug delivery

## Abstract

Mucin networks are viscoelastic fibrillar aggregates formed through the complex self-association of biopolymeric glycoprotein chains. The networks form a lubricious, hydrated protective shield along epithelial regions within the human body. The critical role played by mucin networks in impacting the transport properties of biofunctional molecules (e.g., biogenic molecules, probes, nanoparticles), and its effect on bioavailability are well described in the literature. An alternate perspective is provided in this paper, presenting mucin’s complex network structure, and its interdependent functional characteristics in human physiology. We highlight the recent advances that were achieved through the use of mucin in diverse areas of bioengineering applications (e.g., drug delivery, biomedical devices and tissue engineering). Mucin network formation is a highly complex process, driven by wide variety of molecular interactions, and the network possess structural and chemical variations, posing a great challenge to understand mucin’s bulk behavior. Through this review, the prospective potential of polymer based analogs to serve as mucin mimic is suggested. These analog systems, apart from functioning as an artificial model, reducing the current dependency on animal models, can aid in furthering our fundamental understanding of such complex structures.

## 1. Introduction

Mucin networks are viscoelastic lubricious layers, lining the luminal surface of many regions within the human body. The network formation results from a continuous deposition of biopolymeric glycoprotein chains. Through the structural, chemical and barrier properties, these functional coatings serve a critical role in maintaining human health. When these coatings are lost, a perceivable dryness along the cornea (dry eyes), buccal cavity (xerostomia) or upper respiratory tract can occur, rendering the epithelium highly vulnerable to pathogens (e.g., intestinal inflammation from loss of gastrointestinal mucus). Despite mucin’s importance, it is often simply considered a barrier to be overcome, for instance, its role in affecting drug permeability and therapeutic bioavailability. Here, we present a detailed review highlighting the unique structure and functional role that mucin plays and also present avenues by which mucin considerations can be built into biomaterial/biomedical designs, including the capability of synthetic polymeric mucin analogs in the potential therapeutic framework.

## 2. Natural Mucin Network Structure, Formation and Molecular Properties

### 2.1. Molecular Properties

Mucins are glycoproteins secreted by epithelial goblet cells, and mucus cells present in submucosal glands [1]. For instance, in the oral mucosal surface, the acinar epithelial cells, and in gastric region, the foveolar cells are primarily responsible for mucin production. These mucin glycoproteins are key constituents comprising the lubricating layer, and in the oral cavity, they primarily determine the rheological properties of saliva [2,3,4]. These glycoproteins can be broadly categorized on the basis of their molecular weight (*M*_w_) and degree of interconnection. In the oral cavity, the low *M*_w_ glycoprotein chains of 200–300 kDa, made of a single glycosylated peptide chain, form an important fraction of free flowing saliva and they help regulate bacterial clearance. While, the high *M*_w_ glycoproteins of >1000 kDa constitute a significant part of oral salivary films, or mucin network coats (bound saliva), which helps form a protective epithelial shield [5,6,7]. In the buccal region, these functional coats are more commonly referred to as oral mucus, mucin coats or the salivary pellicle [2,3,4].

**Figure 1:**
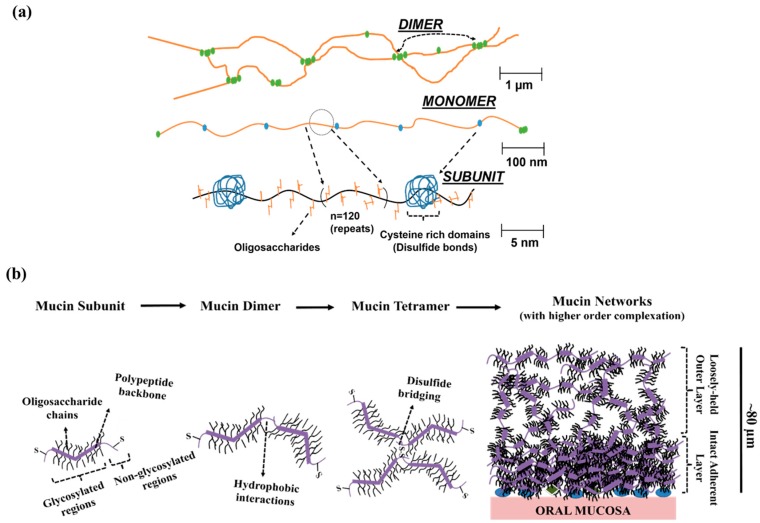
Mucin glycoprotein’s molecular properties, and its network formation ability. (**a**) A simplified scheme shows the composition of mucin glycoproteins, its subunit, monomer, and dimer; (**b**) Progression of higher order complexation of mucin glycoproteins resulting in the formation of a mucin network over oral mucosal surface. This scheme demonstrates the progression of high-order complexation process, which results in formation of mucin aggregates. Mucin aggregates invariably contain two-distinct zones: the more intact adherent mucin layers, and loosely-held (expanded) mucin layers of high free-volume. The illustrated scheme is adapted from References [22,23,24].

In general, mucin contains a polypeptide backbone which is predominantly made of serine, alanine, proline, glycine and threonine (~75% of total amino acid content) [8,9,10]. The oligosaccharide side chain decorates the peptide backbone, which are covalently grafted via *O-*glycosidic linkages (Figure 1a). The linkages were formed between hydroxyl groups in peptide backbone (e.g., serine, threonine) to the sialic acid or l-fucose subunits present in oligosaccharide chains [10,11,12]. The *M*_w_ of the hydrophilic oligosaccharide side chains are highly polydisperse, and vary substantially between species and mucosal surfaces, and constitute nearly 50%–80% of mucin’s dry weight [11,13]. These carbohydrate chains are composed of the monosaccharide subunits, galactose, sialic acid, which renders mucin with hydrophilic properties [11,12,13]. Mucin chains also contain non-glycosylated regions that are rich in cysteine moieties, enabling interchain disulfide crosslinking. The non-glycosylated regions are vulnerable to proteolytic cleavage due to lack of oligosaccharide chains [12]. Thereby, under proteolytic conditions, the mucin chains can break-down into smaller subunits. In oral environment, such mechanism aid in forming the free-flowing saliva [4,14].

Mucin possesses a bottlebrush-like structure. The high molecular weight glycoproteins are structurally analogous to filaments possessing a high structural aspect ratio (L/D) [15]. These glycoproteins crosslink via disulfide bonds, and forms longer branched structures (mucin multimers). Subsequently, the multimers can randomly undergo higher order associations with glycoprotein chains and with small molecule proteins (Figure 1b). These associations are driven by a wide variety of molecular interactions such as disulfide bridging, hydrophobic interactions, electrostatic forces and hydrogen bonding, forming a more intact, 3-dimensional viscoelastic biopolymeric network [8,16]. For instance, in the oral cavity, the salivary proteins adsorb onto the mucosal epithelial surface. The process is accompanied by continuous deposition of glycoproteins chains (self-association) and complexation with oral proteins [4], forming oral mucus coats.

### 2.2. Mucin Network Structural Characteristics

Morphologically, in the initial formation stage of a developing mucin coat, the glycoprotein creates granular inhomogeneous deposits. However, at greater time scales, due to prolonged fibrillar depositions and a complexation process, those granular structures merge into a continuous interwoven morphology (reticulated structures) [17,18]. For instance, Lie *et al.*, (1976) [18] used hydroxyapatite/epoxy resin splints as a model enamel substrate in studying the mechanism of mucin pellicle formation. In their study, the splints were bound to the buccal surface. At relatively short time scales (~2 h), the pellicles formed heterogeneous granular structures with a thickness ranging from 25 to 125 nm. While at greater time scales (~24 h), they formed a continuous thick film of ~550 to 900 nm [17,18,19,20]. In a more recent study, Baek *et al.*, (2009) used optical approaches to study the time-dependent salivary pellicle formation over teeth, in agreement with the earlier results, this study suggested a similar structural surface growth tendency [21]. Interestingly, such growth can also be observed in multilayered particulate based systems. For instance, in our recent work [15], we demonstrated that by utilizing a filamentous micellar structure analogical to mucin glycoproteins, a similar growth tendency can be recreated via affinity driven layer-by-layer deposition approach. The surface growth tendency during micellar deposition follows a lateral-expansion mode, initially forming localized islands. These clusters, due to the high aspect ratio of filomicelle, easily bridged with each other to form reticulated structures similar to mucin. Similarly, Hong *et al.*, (2005) in their study suggested that micellar aggregates possess close semblance with mucin aggregation tendency [20].

In a more simplistic representation, mucin networks can be visualized as multilayers of mucin glycoproteins. In the respiratory or gastrointestinal tract, the networks contain two distinctive zones, (1) a loosely adherent outer layers with an expanded free volume that are prone to easy removal; and (2) a denser, more intact, mucosal adherent inner layer [25,26,27,28,29]. The loosely held outer layers are formed via disulfide intermolecular bonding. While, the inner layers of mucin networks are self-organized from relatively longer mucin chains, whose hydrophobic domains firmly anchors it to the epithelial surface. Further, aided by complexation, they form a more robust, tight network [24].

Mucin network’s structure and its rheology can be highly interdependent, which can be impacted by localized pH and ionic concentration. For instance, using gastric mucin, studies by Cao *et al.*, (1999) [30], which was later supported by Hong *et al.*, (2005) [20], have demonstrated that mucus undergoes pH-dependent sol–gel transition, where at simulative gastric environment (pH < 4), the glycoprotein chains possess an extended conformation as opposed to the random coil state. This chain reorientation favors the hydrophobic complexation between glycoprotein chains, causing the gelation of a viscous solution into a tight protective intestinal barrier. Apart from pH, such conformational shifts can occur due to changes in ionic environment effecting from electrostatic charge interactions, causing swelling or shrinkage of bulk networks [30]. Hence, influenced by regional environmental conditions, the structural organization of natural mucin networks can differ regionally and temporally throughout the body. This structural property variation (e.g., pore size, viscosity) plays a critical role in its biofunctional behavior. As an example, human cervical mucus shows changes in its network structural organization based upon the phases of the menstrual cycle, impacting the spermatozoal transit [31,32]. It should be noted, this structural-biofunctional interdependence in mucin networks can be governed by the composition of the type of mucin, which in turn is associated with subjects health condition (healthy *vs.* diseased state). For instance, studies by Thornton and co-workers [33] have established that in respiratory mucin networks, its oligomeric mucin composition (MUC5AC, MUC5B) shows variation between diseased (e.g., cystic fibrosis, chronic obstructive pulmonary disease (COPD)) and healthy subjects.

**Figure 2:**
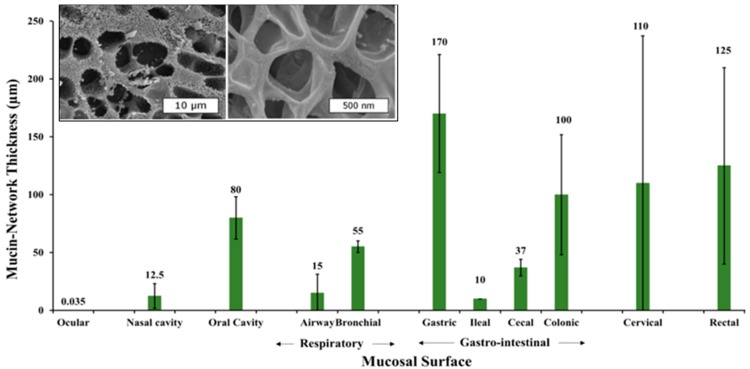
Mucin network thickness varies based upon its physiological location and role. Based on findings from References [39,40,41]. Inset figure shows cryo-SEM imaging of pulmonary mucin, demonstrating heterogeneous mesh size distribution. Reprinted with permission from Reference [42]. Copyright © 2009, National Academy of Sciences.

The radius of gyration of high *M*_w_ natural mucin (~19,000–32,000 kDa) ranges from ~190 to 270 nm. In a more detailed look, the mucin diameter can range from 3 to 10 nm, and its length can span out from 100 nm to several microns [34]. For instance, studies by Round *et al.*, (2002) [35] have shown that in ocular regions, the glycoproteins contour length distribution can extend up to 1500 nm [35,36]. These glycoprotein chains self-organize forming networks, whose structural properties such as thickness and mesh size, can vary significantly both within and across different regions (inset Figure 2). For instance, in human gastrointestinal track, the mucin network thickness is found highly variable ranging between 50 to 450 µm (see Figure 2 for thickness distribution in different mucosal surface) [37]. Likewise, the thickness variations can also be seen in different species, for instance Atuma *et al.*, (2001) [25] measured the rat gastro-intestinal mucin network thickness. In their studies, the network thickness was quantified for both intact and loosely-bound mucin layers. Recent work by Ermund *et al.*, (2013) [38], illustrated that in mouse models, the small intestine possesses only the loosely held mucin layers, thereby facilitating easier nutrient absorption. However, in large intestine, the mucus layers are thicker and contain both the loosely held and intact layers, where its inner bound layers functions to selectively block the bacterial access into epithelial surface. This exemplifies the interrelated structure-functional dependency in natural mucin structures.

The thickness variations in mucin networks is highly dependent on their proximity to the secretory glands and other physical factors, such as breathing, mastication, and swallowing can also significantly affect [39]. For example, studies by Collins *et al.*, (1987) [39] and Tinanoff *et al.*, (1976) [43] have compared oral mucin thickness in adult and children (ranges ~50–100 µm), and found it to be independent of variations in the total available surface area. However, relative to buccal soft tissue, the mucin thickness along enamel surfaces is significantly lower (~0.03–0.1 µm), suggesting variations along different regions.

The mucin structure can also possess a high degree of heterogeneity in its mesh size, both within and across different regions. For instance, study by Olmsted *et al.*, (2001) [40] on human cervical mucins have suggested its pore size to range between 20 to 200 nm. Similarly, Lai *et al.*, (2011) in their work reported that in cervicovaginal regions, its mesh size can range between 50 to 1800 nm [44], while Matsui *et al.*, (2006) [45] have reported that in tracheobronchial mucus, they range from 200 to 1000 nm.

## 3. Natural Mucin Network Function

**Figure 3:**
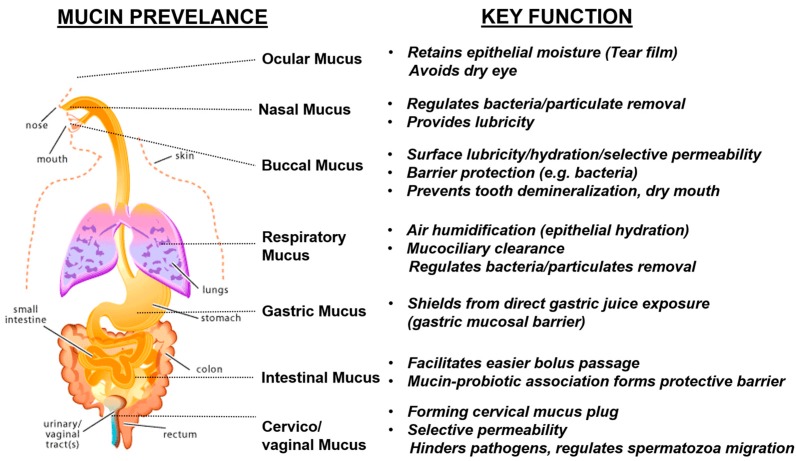
Illustrative scheme highlighting mucin network prevalence and its key functional properties across different regions within human body. The respiratory-gastrointestinal figure outline was taken from Reference [46].

Mucin is almost ubiquitously presently in all biological systems. They line the mucosal epithelium in the ocular, nasal, buccal, respiratory, gastro-intestinal tract, vaginal and rectal regions. In general, mucin networks regulate bacterial adherence (aggregation and/or clearance), provide surface lubricity, retain moisture (through surface hydration), acts as a protective shield for the underlying mucosal surface, and allow for a selective permeability (e.g., oxygen transport, nutrients) (Figure 3) [4,5,6,7].

In eyes, the protective barrier is referred as the tear film (thickness ~6–10 µm) [13], which is comprised of (1) an innermost ocular mucin coat that immediately surrounds the epithelial surface in protecting them; (2) an intermediate aqueous layer that is formed from soluble mucin components; and (3) an outermost oily layer. The function of the mucin layer in tear film is to help (1) regulate the stability of aqueous-oily layers [47]; (2) act as a barrier in protecting the underlying epithelial surface; and (3) aid in retaining epithelial moisture [47,48,49]. Interestingly, in subjects with contact lenses (e.g., silicone hydrogel lenses), upon prolonged wearing, these ocular mucin coats can roll up into spherical balls (mucin balls). Despite this change in morphology, these spherical balls do not appear to impact the visual responsiveness, cause discomfort, or contribute to bacterial invasion [50]. Some recent work has even suggested the potential role of mucus balls in obviating corneal infiltrative events (e.g., corneal inflammation), associated with contact lens wear [51].

In the buccal cavity, the mucin networks offer selective permeability (e.g., unhindered transport of solubilized molecules to taste receptors), and coupled with its barrier properties (e.g., lubrication, surface hydration and protection), helps regulate oral health. Clinically, with a lack of these functional properties, oral dryness can occur, (e.g., xerostomia) accompanied by painful inflammation. In United States, one to four million people suffers from Sjőgren’s syndrome (dry mouth & dry eyes) and nearly 20%–30% of the people were affected by xerostomia with even more prevalence among senior and women [52,53,54]. This hyposalivation may be temporary among certain populations, but with patients undergoing treatment such as radiotherapy, they are at a high risk of developing an irrecoverable damage to salivary glands, causing permanent oral dryness. In xerostomic patients, there is an increased susceptibility to microbial colonization leading to oral infections (e.g., candidiasis) [52,54,55]. For instance, mucin contains antifungal peptides (e.g., histatins, MUC7 12-mer) [56,57,58,59], which play a key role in inhibiting candidiasis, a prevalent fungal infection that results from Candida albicans. Nearly 95% incidence rate of candidiasis was reported among patients with acquired immunodeficiency syndrome (AIDS) [60]. Wide clinical research were performed in an effort to understand the effects of oral salivary flow rate and/or its composition on pathogenesis of candidiasis. Their studies showed nearly 37%–40% reduction in oral salivary flow rate among HIV+ patients [60,61], and the antifungal mucin components in salivary compositions were also found significantly lower [58,62], culminating into a diseased condition.

Mucin networks, when extended over enamel surfaces, are referred to as acquired enamel pellicle. When the tooth surface is exposed to acidic conditions, the pellicle layer aids in reducing enamel-demineralization [19], Mechanistically, the mucin networks are formed through higher ordered complexation-aggregation deposition process as that of acquired pellicles, independent of the substrate-interfacial site (enamel or buccal surface) or tissue hardness (soft or hard oral tissue).

In the respiratory tract, the mucin network regulates bacterial and particulate removal through mucociliary clearance. During inhalation, the respiratory mucin coats provide air humidification and helps maintaining epithelial hydration [63]. In regions of the stomach and duodenum, the adherent mucin layer acts as a shield, protecting the epithelium from direct gastric juice exposure (e.g., hydrochloric acid, pepsin), commonly referred as gastric mucosal barrier (GMB) [64]. The barrier property in GMB is due to the formation of a mucin–bicarbonate barrier, where the bicarbonate secretions occur from the mucosal surface. This barrier creates a pH gradient across luminal (low pH) to epithelial side (high pH), protecting the epithelial surface [65,66]. Also, the mucin networks are selectively permeable, allowing for cell migration and repair of damaged tissue. The mucin network creates a micro-environment for cellular components (e.g., cells, plasma proteins), forming a mucoid cap via its scaffold-like structures, thus promotes revascularization [37,41].

In the gastrointestinal tract, mucin’s lubricative effect facilitates an easier passage of the food bolus through the intestine. In addition, mucin serves as a matrix that promotes adherence of probiotic strains (e.g., lactobacilli, bifidobacterium) [67,68,69]. The synergistic probiotic–mucin association helps form mucosal barriers, protecting against harmful microbial infections (e.g., bacterial, viral) [44,67,70,71,72]. Any compromise in the barrier integrity could alter food digestion, render epithelium vulnerable to enteric pathogens and can cause intestinal inflammation [68,73].

**Figure 4:**
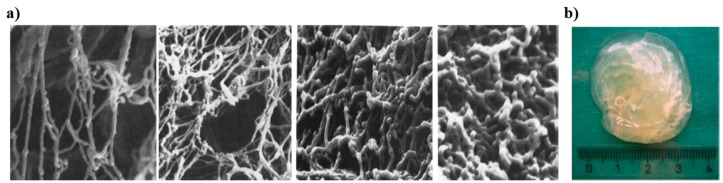
(Left) CMP at different stages of pregnancy (**a**) (left to right) at 20th day, 5 weeks, 2-1/2 months, and last week of pregnancy. Cervical mucin network density increases progressively during the gestation period, and forms a more compact interconnected fibrillar networks. The mucus functions as a selectively permeable plug that blocks pathogens and allows nutrients and growth factor to transport. Reprinted with permission from [80]. Copyright © 2011, John Wiley & Sons, Inc.; (**b**) Figure showing bulk structure of cervical mucus plug discharged during labor. Reprinted with permission from [77]. Copyright © 2010, John Wiley & Sons, Inc.

In the cervix, the mucin network functions like a selective permeable layer, commonly the cervical mucus plug. The mucus plug hinders transport of bacteria and viruses (e.g., *Lactobacillus* spp., herpes simplex virus [74,75]) in the reproductive tract, while preferentially aiding the motility of spermatozoal migration (Figure 4). Any irregularities in function of the cervical mucin networks can even cause infertility [76,77]. A recent study has demonstrated the protective barrier effect of human cervicovaginal mucus in impeding HIV type1 transport to underlying epithelium [78,79].

### 3.1. Mucin Network’s Role in Microbial Regulation

Mucins, via their adhesion tendency, play a critical role in microbial capture across different regions, which helps regulate microbial growth and maintain health. Human body is rich in diverse variety of bacterial flora. For instance, it is estimated in humans that nearly 500 species of bacteria populate the oral cavity (e.g., *Streptococcus*, *Veillonella*) [81,82,83,84]. In the epithelial surface, the loosely held mucin outer layers can function as a sacrificial trap in bacterial adherence, which coupled with oral salivary flow mechanism can help regulate the microbial clearance [8]. However, since the innermost mucin layers forms a robust tight barrier, they function as a protective shield, preventing bacterial transport to the underlying epithelia. For instance, in the gastrointestinal tract, the mucin barriers shields the epithelium from polymicrobial infection. This barrier function, if compromised, can lead to chronic inflammation, causing ulcerative colitis [68]. The mucin bacteria adherence mechanism can be more readily understood by studying the bacterial growth process over tooth surfaces. The mucin pellicles promote bacterial adherence, yet also helps protect the tooth from acid demineralization effects [85]. Interestingly, if the adherence process is left unregulated, this can result in serious complications, including dental caries and periodontal disease or vocal disorder due to laryngeal mucus [86], cystic fibrosis (CF), COPD or bronchial asthma in pulmonary tracts [87,88]. Study by Matsui *et al.*, (2005) [89] have suggested the due to unregulated thick mucus secretion from cystic fibrosis, there is a reduced migration ability of neutrophils across the bacterial trapped mucus barrier, thereby compromises neutrophil’s bacterial killing ability and contributes to airway infections.

Extensive studies were performed to understand the bacterial distribution among different mucosal regions, and how their diversity changes with pathologic conditions. For instance, Aas *et al.*, (2005) studied the bacterial flora diversity in different regions (hard/soft tissues) of the oral cavity, and found that there is significant difference in bacteria flora distribution between diseased and healthy patients [83]. The adherence tendency of mucin also helps form a desirable physical barrier that can hinder permeation of virus. For instance, Lai *et al.*, (2009) [74] found that in cervicovaginal mucus, the transport rate of herpes simplex virus (~180 nm) was atleast 8000 fold lesser than the non-mucoadhesive carboxyl-modified polystyrene particles (~200 nm), illustrating mucins less emphasized role as protective shield.

The mucin-microbial capture mechanism can be attributed to the specific surface receptors available in the microbes (e.g., adhesins such as lectins and enzymes). Driven through non-covalent molecular forces such as charge and hydrophobicity (mucoadhesive effect) [2,90], the microbial receptors specifically bind to the oligosaccharide chains available in mucin glycoproteins (e.g., oral pellicle) [2,83,91,92,93]. During the mucoadherence process, the favorable molecular conformation of glycoprotein chains facilitates for an improved microbial capture. For instance, mucins with high proline content, due to its hydrophobic nature, easily anchors to the epithelial surface, which allows oligosaccharide side chains to orient toward exterior apical side, aiding adherence [8,94,95]. Apart from adhesive forces, the screening effect emanating from the size differences between network mesh and microbes can also play an important role, impacting microbial accessibility to the underlying surface.

## 4. Mucin Networks as Physical Permeation Barrier for Biofunctional Molecules

**Figure 5:**
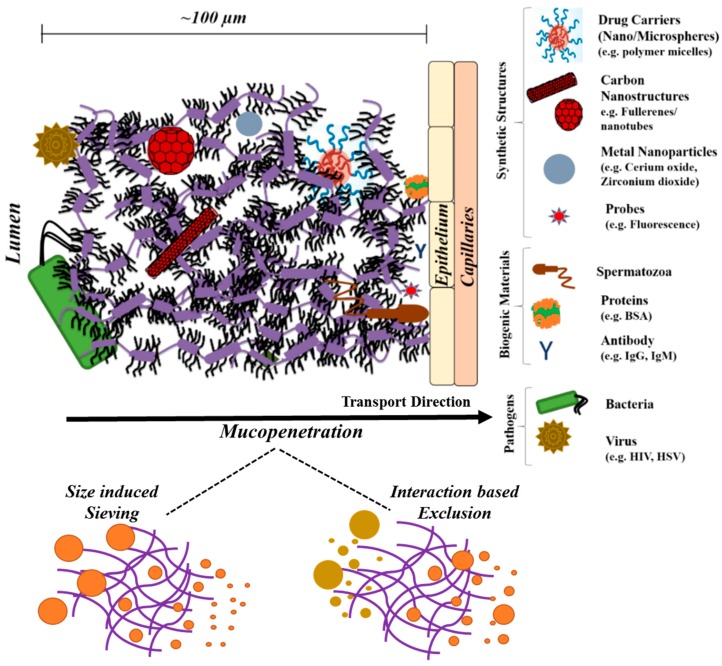
Schematic of mucin network’s (cervical region) ability to impact transport properties of various biogenic materials, synthetic structures or pathogens across its physical barrier. Figure also illustrates the mucopenetration effects, which can arise as a result of differences in network mesh size, or its adhesive interactions with transport molecules. Figure was adapted from References [105,106].

In general, mucin networks allow for selective permeability. Mucin glycoprotein impact the diffusional properties of agents through steric obstruction or adhesive interactions (e.g., hydrogen bonding, hydrophobic and/or ionic interactions) or size exclude based on its mesh spacing (Figure 5). Such reduced permeability of biofunctional molecules (e.g., drugs carriers such as nanoparticles) can have an effect on therapeutic bioavailability in the underlying mucosal epithelium. Efforts to minimize nanoparticle interactions (mucoadherence) with mucin barriers have received significant attention, due to the recent advancements in the field of nanomedicine. The hindered transport rate in many of the nanoparticle based systems can emanate from adhesive interactions with the mucin chains [96]. Studies have well-articulated that nanoparticle charge properties can play an important role during the trans-mucus permeation [97,98]. In improving mucopenetration, a near neutral charged nanoparticle was preferred (Coaker (2014) [97]). Such charge tendency is not limited only to nanoparticle based systems. Recently, Ribbeck and co-workers (2013) [99] have demonstrated a reduced permeability rate for both cationic and anionic peptide transport across mucin barrier. In their study, apart from net charge, the spatial charge distribution of the transport molecule and ionic environment of mucin barrier were found to play a crucial role in affecting the transport kinetics within mucin.

Commonly, permeation based studies were performed using FRAP (fluorescence recovery after photobleaching), or particle tracking technique such as single/multiple particle tracking, time-resolved confocal microscopy or fluorescence correlation spectroscopy (FCS) [79]. For instance, in gastrointestinal mucin networks, Crater *et al.*, (2010) [98] have demonstrated that a significantly reduced mobility can be observed among polystyrene nanoparticles with different surface charge modifications (amine, cationic and sulfate) via multiple particle tracking measurements. Mucin networks form a protective shield, which hampers mobility of most the metal nanoparticles (e.g., cerium oxide, Zirconium dioxide) and can immobilize carbon nanotubes (Jachak *et al.*, 2012) [96].

In improving mucopenetration, the use of PEGylated stealth nanocarriers has been attempted. Such PEGylation approaches are commonly utilized in improving drug circulation time from reduced opsonization effects. Due to such added prevalence, studies pertaining to transport behavior of PEGylated nanocarriers across mucin barrier have received considerable attention in recent years. For example, Wang *et al.*, (2008) [100] have shown that among PEGylated nanocarriers, the extent of PEG surface coverage and its molecular chain length can significantly impact the nanoparticle interpenetration through cervical mucus. By increasing the PEG chain length, there is an increased probability for its entanglement with mucin glycoprotein chains, thereby can entrap/reduce the carrier transport rate. Also, even with lower *M*_w_ PEGylation, by increasing the net negative charge or reducing the degree of PEG surface coverage can result in a hampered diffusional rate [100].

In general, the small molecule proteins like bovine serum albumin and antibodies (e.g., immunoglobulins IgG, IgA and IgM) [101] possess weak interaction with glycoproteins chains, and owing to its considerable size difference with mucin mesh, their transport rate were only weakly hindered [40,101]. Due to similar size-based effects, smaller viruses can diffuse less hindered through mucin barriers. However, with antibody (Ab) bound pathogens (e.g., Ab-virus such as HIV [102], HSV [103]), the permeation can be hampered from increased adhesion effects from multiple low affinity antibody-mucin interactions [104].

## 5. Critical Role of Mucin Network in Impacting Diverse Bioapplications

### 5.1. In Designing Drug Delivery Systems

Traditionally mucin is viewed as a barrier to overcome for drug delivery applications. For instance, mucin as a physical barrier can limit drug permeation, and efforts to improve the drug carrier stealth tendency by varying its physical (e.g., size) or chemical (e.g., However, by understanding the mucin network functional properties, it proposes wide potential in variety of bio applications.

For instance, the mucin’s adherence tendency can be used beneficially in designing drug delivery systems (DDS) such as drug encapsulated microsphere/nanoparticle formulations, or mucoadhesive gels/tablets/patches. Recent findings [34,107] suggest that mucin networks can immobilize most of nanomaterials (e.g., polymeric nanoparticles) or alter its particulate transport via adhesive and steric forces. For gastro-intestinal (GI) drug delivery, such mucoadhesive effects can be used to enhance the drug transit time across GI tract. The use of mucoadhesive polymers (e.g., chitosan, hydroxypropylmethylcellulose (HPMC), poly(vinyl pyrrolidone) (PVP), poly(ethylene glycol), carboxymethylcellulose (CMC)) [108,109,110] has been shown to increase the residence time, thereby improving the efficacy of drug adsorption.[34,111,112,113,114,115] Araújo *et al.*, (2014) [115] have demonstrated that in chitosan coated nanocarriers (e.g., poly(lactide-*co*-glycolide), solid lipid nanoparticles and porous silicon), a more sustained release of payload (e.g., glucagon like peptide-1) with enhanced bioavailability resulted from its mucoadherent tendency. Thus, these DDS can be utilized in ocular [116,117,118,119], buccal [120,121,122,123], nasal [124,125,126,127], pulmonary [128,129,130,131], vaginal [132,133,134,135,136], or rectal [137,138,139,140] delivery, for eliciting a therapeutic effect either locally or systemically [13,114,141].

### 5.2. Mucin as a Bio-Functional Coat

Recently, mucin has been studied for its potential as a functional coat to inhibit biofouling and promote cell pattering. For instance, Thasneem *et al.*, (2013) [142] have shown that mucylated nanocarriers (e.g., polylactic-*co*-glycolic acid) possess reduced opsonization effect with promising haemo- and cyto-compatibility. Mucin also possesses interesting potential when utilized as an implant coating (e.g., vascular grafts), offering excellent surface lubricity and haemocompatibility. For example, Janairo *et al.*, (2014) [143] demonstrated in rat models that implants containing covalently grafted mucin layers can effectively inhibit platelet adhesion and prevent thrombosis. In their studies, the untreated vascular grafts showed higher platelet adherence (~210 platelets/cm^2^), however upon mucin incorporation the surfaces exhibited significantly reduced platelet adhesion (<60 platelets/cm^2^). The mucins can be included either as a passive adsorption (~60 platelets/ cm^2^) or can be covalently bound (PEGylated mucin) (<10 platelets/cm^2^) to those implant surfaces.

Mucin coatings can also reduce protein and neutrophil adherence onto biomaterial substrate like polyethylene terephthalate, which can be beneficially used in reducing biomaterial mediated inflammatory host response [144]. Similarly, studies by Shi *et al.*, (2000) [145] have demonstrated mucin’s ability to improve surface wettability and inhibit bacterial adhesion over variety of synthetic surfaces such as polystyrene, polyurethane, silicone, and poly(methylmethacrylate). Such microbial inhibitory and lubricative function provides exciting opportunities for its use as coatings in various biomedical devices (e.g., stents, catheters), that require high surface lubricity and low friction.

**Figure 6:**
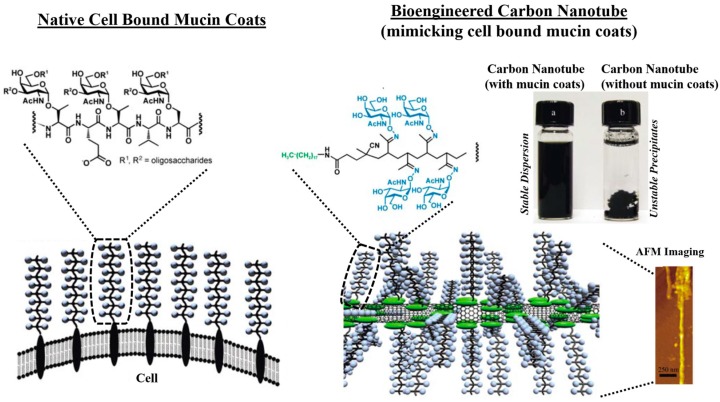
A comparison of native cell bound mucin coats with biomimetic carbon nanotubes possessing cell bound mucin coats. The bioengineered carbon nanotubes mimics the native cell bound mucin functional property, and can be used in preventing non-specific protein binding (antibiofouling), or immobilizing specific functional molecules to CNT via specific biomolecular recognition with mucin chains, or as coatings for developing stable dispersions. Reprinted with permission from Reference [148]. Copyright © 2004, Wiley-VCH Verlag GmbH & Co. KGaA, Weinheim.

In a more recent work, Crouzier *et al.*, (2013) [146] have demonstrated that mucin can serve as an elegant cell-repulsive biopolymeric coat, which can be used in regulating adherence of different mammalian cell types (e.g., epithelial cells, fibroblasts, and myoblasts. While, Johnson *et al.*, (2009) [147], in their work have functionalized peptide microtubes with mucin glycoproteins, which demonstrated improved cell adhesive property (normal rat kidney cells) and allowed for cell spreading along its aggregated microtubular structures. Such mucylation of micro- or nanotubes with drug encapsulation can be used for targeted payload delivery and improving the drug bioavailability. As a surface modifier, Bertozzi and co-workers (2004) [148] have demonstrated that glycosylated biopolymers grafted to carbon nanotubes (CNTs) mimics the natural mucin arrangements present along the cell surface (Figure 6). Such modified CNTs can inhibit non-specific protein adsorption.

Mucin possesses an interesting complexation capability with hydrophobic molecules, which can improve its aqueous solubility. Drug *et al.*, (2010) [149] in their work demonstrated mucin (bovine submaxillary mucin, -BSM) was able to complex with polyaromatic hydrocarbons (e.g., Benzo[a]pyrene). These mucin-complexed molecules demonstrated an improved bioavailability and membrane-penetration ability. Likewise, Gozin and co-workers (2010) [150] demonstrated that bovine submaxillary mucin can stabilize dispersions of hydrophobic nanocolloids such as C60 fullerene, inorganic fullerene-like gastro-intestinal (IF-WS_2_) and multi-walled carbon nanotubes (MWNT) in aqueous solution.

### 5.3. Mucin in Drug Delivery and Tissue Engineering Applications

The critical role of mucin as a functional scaffold is relatively less emphasized in natural settings. For instance, studies have demonstrated that mucin can associate with carbohydrate-binding proteins like selectin and lectin [151,152,153,154]. While, during the early phases of pregnancy (peri-implantation period), cervical mucus scaffolds can impact adherence of blastocysts possessing l-selectin rich outer walls, and thereby critically affect embryo attachment to the uterus [155].

Inspired by such capable mucin-protein interactions, recent studies have demonstrated the ability to synthetically develop versatile multilayered structures/scaffolds via layer-by-layer (LBL) approach, for potential drug delivery and regenerative applications. For example, Crouzier *et al.*, (2012) [156] and Polak *et al.*, (2014) [157] have shown that structurally robust multilayers can be fabricated from mucin sugar–lectin interactions. Mucin glycoproteins were rich in sugars moieties such as sialic acid and *N*-acteyl-d-glucosamine [157,158], which are specific towards lectin moieties. The multilayers formed via those specific linkages are highly stable, and can remain unaffected by effects of salt or pH shifts [156]. Utilizing the specific mucin-lectin linkages, previously studies have been demonstrated in drug carriers (e.g., microspheres) systems, where particulates can be surface functionalized with concanavalin-A (lectin) in developing a targeted drug delivery systems [159,160].

Apart from carbohydrate-protein interactions, mucin-based multilayers can also be developed via conventional charged based interactions. Mucin glycoproteins possess net negative charge, hence utilizing its ionic tendency, mucin can be self-assembled along with a positively charged polymer (e.g., poly(diallyldimethylammonium chloride) [161], chitosan [162]) using LBL deposition technique. Interestingly, Wang *et al.*, (2012) [161] in their recent work used pig gastric mucin (PGM) and poly(acrylamide-*co*-3-acryl-amidophenylboronic acid) (P(AAm-AAPBA)) in developing a stimuli responsive multilayered scaffold. The self-assembling process was driven by formation of boronate ester linkages between polyols in PGM with boronic acid units in P(AAm-AAPBA). Those linkages are reversible under glucose environment, and the multilayers formed can be selectively disassembled and utilized for stimuli induced drug release [161,163,164].

In regenerative medicine applications, mucin-containing artificial saliva (e.g., Saliva Orthana^®^) can be used as an effective salivary substitute. Various clinical studies are performed and have corroborated mucin’s beneficial role in developing buccal formulations [165,166,167,168]. Mucins through its hydrated topical coat helps alleviate oral mucosal complications such as xerostomia and mucositis [169,170]. For instance, study by Davies *et al.* (1998) [171], showed nearly 73% of xerostomic patients showed improvement through use of mucin based salivary substitutes, and work by Sweeney *et al.*, (1997) [172], from their clinical trials suggested excellent patient compliance, when administered as an oral spray.

In a recent work from Duffy *et al.*, (2015) [173], they used methacrylated mucin to form a covalently crosslinked hydrogel. These mucin-based scaffolds are capable of loading both hydrophobic and hydrophobic drugs, via its intrinsically diverse chemical nature (hydrophilicity) and its mucoadhesive interactions (e.g., hydrophobic, electrostatic forces), and was demonstrated for its ability to offer a sustained drug release.

## 6. In Modeling Importance and Need for Rigorous Characterization

In drug delivery applications, the mucin coats and its underlying epithelium were the main contributors to reducing drug permeability across mucosa surfaces [34,174,175]. In an effort to improve the drug permeability across the mucosal barrier, there is a growing interest among researchers to understand the network properties (physical and chemical behavior). In understanding mucin network barrier properties, Gniewek *et al.*, (2012) [176] used a coarse-grained model (polymer lattice model) to study nanoparticle transport through mucin networks. Their results suggested that the innermost layers in mucin networks are firmly held and play a critical role in hindering the particle diffusion. However, due to the mucin network formation complexity, it poses a significant challenge to understand its interrelated structural and chemical properties on its bulk network behavior [177].

Much of the recent work have centered on studying the effect of nanoparticle physicochemical characteristics (e.g., nanoparticle size [178], surface chemistry [40,179,180], and PEGylation [178]) on the transport properties across mucin networks. For example, Norris *et al.*, (1996) [179], used polystyrene nanoparticles in studying the effect of particle size, surface hydrophobicity and charge properties on transport across gastrointestinal mucus. Their study suggested that there is a significant drop in permeability when particle diameter reaches ~300 nm (permeability ~5 × 10^−5^ cm/min), and a more gradual drop in its transport rate for concomitant increase in particulate diameter till ~500 nm (permeability ~0.7 × 10^−5^ cm/min). Additionally, their study suggested that compared to ζ-charge potential the surface hydrophobicity of particle can play a critical role in hindering the particle translocation. Thus, by increasing the relative hydrophilicity a lesser transport resistance for particle migration was expected.

Despite such significant efforts, the bulk network behavior remains unclear and in some instances, contradictory. For instance, Lai *et al.*, (2007) [178] studied the transport of PEGylated nanoparticle across mucin networks, using NPs of different diameters. To obviate PEG interpenetration with mucin glycoprotein chains, the study utilized relatively lower *M*_w_ PEG chains of ~2 kDa and their results showed an improved transport across mucin network in comparison with uncoated particulates [105,178]. Interestingly, among PEGylated nanoparticles the larger particulates demonstrated a more rapid transport than the smaller particles. In particles with larger diameter ~200, ~500 nm, diffusion was only 6 and 4-fold lower in cervical mucus than that of pure water, respectively. However, for smaller particles (diameter ~100 nm), the diffusional rate was nearly 200-fold lower. This was particularly surprising as the mesh size in the mucin networks was previously reported to be in the range of 10–200 nm [40,101,178].

The formation of mucin networks is a dynamic process, constantly subjected to removal and reformation effects. Thus, a thorough understanding of mucin network properties, its physical and chemical understanding can play a critical role in predicting bulk interaction with drug (or drug nanocarrier) formulations. Such approaches help formulation scientists in designing drugs that allows for a rapid mucin clearance and improved epithelial availability.

## 7. Polymer Network as Synthetic Mucin Analog

### 7.1. Developing Polymer-Based Biofunctional Structures

Developing functional polymeric mimics has received wide attention owing to its application in the areas of regenerative medicine and tissue engineering applications, for instance, the use of hydrophilic three-dimensional hydrogel scaffoldings. However, the polymeric structures, when it replicates the functional and/or structural characteristics observable in nature, becomes a biomimetic analog. Such approaches are not confined only to mimicking complex biomolecules (e.g., protein mimetic polymers, peptide mimics from amino acid conjugated dendrimers) [181,182], but can be developed to translate into a more complex functional analog, such as synthetic mucin networks.

The use of polymer-based biomaterials offers tremendous flexibility, due to its ease in incorporating modifications in its structural, chemical and biofunctional properties, tailored towards a specific application. For instance, through recent advances in polymer synthesis (e.g., atomic-transfer radical polymerization), the polymeric chemical properties can be easily tailored. This in combination with versatile material fabrication approaches (e.g., Layer-by-layer, electrospinning), can be used in forming novel functional structures [183,184,185].

The polymeric building blocks (e.g., di/tri-block copolymers) can also be driven by various molecular interactions such as ionic, hydrophobic/hydrophilic, van der Waals, hydrogen bonding, physical factors (e.g., shape) or micro-phase separation, and can self-assemble to form a more complex architecture [186,187]. In biological applications, such polymeric self-assembled nanostructures are well known for their ability to be biomimic. For instance, inspired by amphiphilic phospholipid bilayers, a considerable amount of interest has been generated in lipid self-assembly and theformation of liposomes [188]. While, in polymeric diblock systems, spherical vesicles (e.g., polymersomes) are comparable to virus capsids [188] and filomicelles are suggested for its structural relevance to filamentous phage [189]. These polymeric structures are derived from directed self-assembly that result from the dissimilar chemical nature of its building blocks. Conceptually, the self-assembly process is analogous to the formation dynamics observable in lipid bilayers, containing distinct hydrophilic head and hydrophobic tail region. These synthetic polymeric structures are well known for their potential as a nano-drug carrier system. For instance, the filamentous micelles possess relatively higher structural aspect ratio than the spherical micellar systems. As a drug carrier, filomicelles exhibited a higher circulation time than the conventional drug delivery systems (DDS).[190] Studies by various researchers (Dalhaimer *et al.*, (2004) [191,192], Discher *et al.*, (2005) [193], Geng *et al.*, (2007) [194], and Shuvaev *et al.*, (2011) [195]) have extensively demonstrated that these filomicelles can be decorated with bio-specific functional moiety (e.g., biotin, antibody) and applied for improving targeting and payload delivery. In a recent study, Pal *et al.*, (2012) [196] used block copolymers of polystyrene-*b*-poly(4-vinylpyridine) (PS-*b*-P4VP) and self-assembled into a hairy nanofibers. These fibrous aggregates were utilized as a structural template for loading gold nanoparticle to form a hybrid metal-polymer construct [196].

### 7.2. Polymeric Network: Role as a Synthetic Mucin Analog

In developing a polymer based synthetic mucin model, Mahalingam *et al.*, (2011) [197] used a stimuli (pH) responsive *in situ* polymeric gels as a synthetic cervicovaginal mucin-mimic. Their study used cross-linkable phenylboronate-salicylhydroxamate based polymeric systems to form networks with increasing crosslinking density from vaginal to seminal pH range (~4.0 to 7.5). The affinity (covalent) paring between boronic acid groups in phenylboronic acid with *cis*-diols allows for reversible condensation that is sensitive to pH changes, allowing for a facile sol-gel rheological transition [198,199,200]. For practical applications, as a cervical coat, in response to seminal fluid, the polymer systems were able to reversibly change from a loosely held viscoelastic to a more robust tighter network. At pH > 4.8, owing to decreased polymeric network mesh size, a significantly hindered virion transport rate was observed (Diffusion coefficient _(Virions)_ ~1.60 × 10^−4^ µm^2^/s at pH 4.8) that was at least one order of magnitude lower in comparison to its transport rate at lower pH. Similar mechanism can be utilized in impacting the migration of macrophages, where in the control placebo gels nearly 250 µm transport of the cell was observed, whereas macrophage diffusion through the custom-developed polymeric gels showed no distinguishable movement. Hence, these synthetic gels can serve as a diffusional barrier to effectively block migration of biogenics like macrophages or inhibit transport of sexually transmittable pathogens like HIV present in the semen. Such polymeric systems apart from biomimetic potential, also provides exciting prospect as a delivery matrix for a localized drug release.

**Figure 7:**
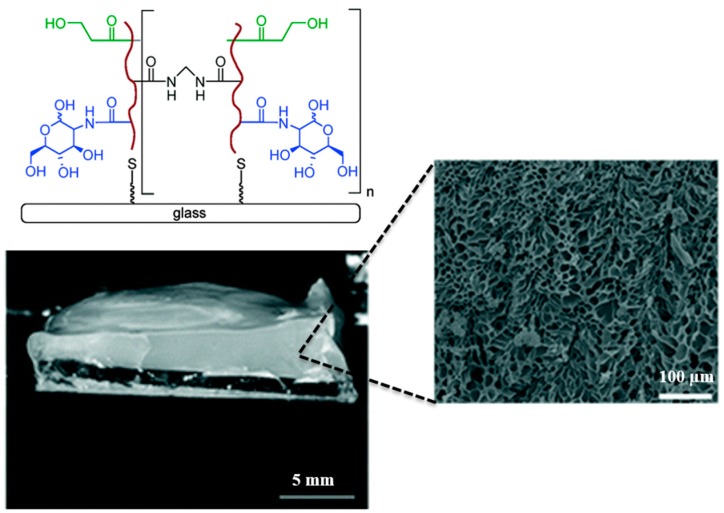
Glass-bound glycopolymer based hydrogels used as a model mucin network mimics in recreating the mucosal surface interfacial property. Scanning electron micrographs clearly shows highly porous structure of the hydrogels, these structures mimics membrane-bound mucin networks that were formed from glycoprotein chains. Reprinted with permission from Reference [201]. Copyright © 2015, The Royal Society of Chemistry.

Similarly, Khutoryanskiy and co-workers (2015) [201], have demonstrated the ability to artificially recreate the mucin’s mucoadherent tendency over glass substrates via formation and use of glycopolymer based hydrogels (Figure 7). The modified surfaces exhibited similar mucoadherent tendency as those found in *ex vivo* animal models. This was demonstrated in their work, through retention of mucoadhesive biopolymers such as chitosan and pectin in the cross-linked hydrogels. Thereby, such approach can be utilized as model framework for studying the mucoadhesive or biological responses (e.g., mucus bacterial capture) that emanates from membrane-bound mucin.

Bertozzi *et al.* [202,203] in an attempt to mimick membrane-bound mucin glycoproteins, synthesized mucin mimetic polymers. Their study utilized polymers such as methyl vinyl ketone, isopropenyl methyl ketone and *N*-[3-(dimethylamino)propyl]-acryl-amide (DAPA), decorated with aminooxy glycans (α-linked *N*-acetylgalactosamine (GalNAc) residues) and additionally end-functionalized with hydrophobic tail groups (e.g., cholesterol, phospholipids, and pyrene). The custom-synthesized synthetic glycopolymers mimicked the rod-like conformation as observed in the natural mucins, and was demonstrated for its ability to remain firmly bound to the supported lipid bilayer due to their hydrophobic anchors. More importantly, their work demonstrated the ability of cell-bound mucins to bind to carbohydrate-specific proteins such as lectin, suggesting molecular specificity and its biomimetic tendency similar to natural mucin. Based on these results, the concept was extended to develop glycopolymer based microarray platforms [203,204,205]. A dual end-functionalized mucin glycopolymers was used, which contain surface anchors tethered to one end and a fluorophore functionalized onto the second terminal. These new classes of glycopolymer were microcontact printed onto azide-functionalized microarray chips, and can be used in probing glycan specific associations (e.g., glycan-specific binding proteins such as agglutinin) [204].

**Figure 8:**
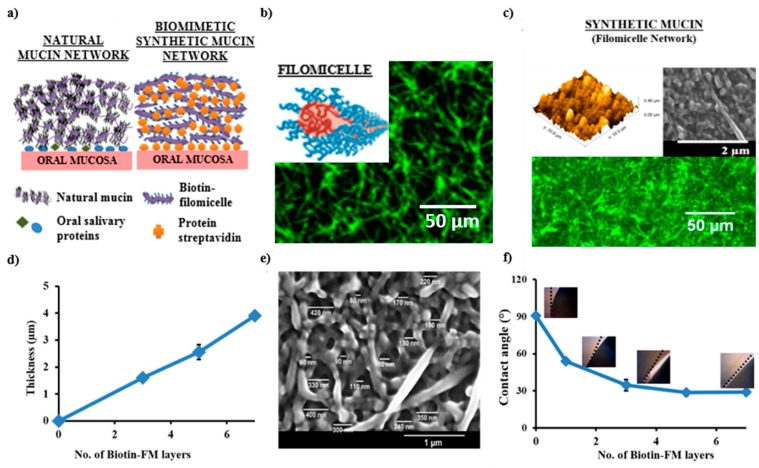
Synthetically derived mucin mimetic system formed from controlled Layer-by-layer (LBL) deposition of polymeric filomicelle, and its structural and functional relevance to natural mucin networks. (**a**) Overall simplified scheme showing natural mucin networks and biomimetic synthetic counterpart (filomicelle networks); (**b**) Fluorescence microscopic visualization of curcumin encapsulated biotinylated filomicelle. Similar to glycoprotein chains in natural systems, the filamentous micelles forms key building block in formation of synthetic networks; (**c**) Morphological relevance of natural mucin structures with synthetic mucin networks formed from filomicelle LBL depositions; (**d**) Controlled thickness growth in synthetic mucin networks can be achieved, by adjusting the number of LBL depositions. Figure shows during filomicelle network, where even with relatively lesser no. of micelle LBL additions (~7), network with significant barrier thickness (~4 um) is achieved; (**e**) Filomicelle networks mimicked the nanoporous mesh size (average range ~110–340 nm) observable in the natural mucin; (**f**) Synthetic mucin networks formed from PEG-based diblock copolymers displayed excellent surface hydration tendency. With synthetic mucin network depositions, the hydrophobic polystyrene (model synthetic interface) translated into a more hydrophilic, hydrating surface, this can be observed with lowering of contact angle from gonimetry study. Reprinted with permission from Reference [84] (Copyright 2015, Wiley-VCH Verlag GmbH & Co. KGaA, Weinheim), and [15] (Copyright © 2014, American Chemical Society).

In a recently published study [15,84,206], it was demonstrated that a controlled layer-by-layer (LBL) deposition of pre-assembled filomicelles crosslinked via biotin-streptavidin affinity interactions, can create a synthetic mucin mimic (Figure 8). In forming synthetic model, PEG–PLA based diblock copolymers were used in forming drug encapsulated polymeric micelles of different shapes (spherical *vs.* filamentous). Structurally, the filomicelles are comparable to natural mucin glycoproteins, due to their high aspect ratio (Figure 8b). Hence, upon surface LBL deposition, these filamentous structures self-organized to form a complex intertwined nanoporous mesh-like structure, resembling natural mucin in its network morphology and mesh size (Figure 8c,e). Interestingly, it was found that the ability to grow into a fused-micelle network is greatly enhanced, by utilizing nanostructures with higher length to diameter aspect ratio like the filamentous micelles as opposed to spherical micelles.

The filomicelle network barrier thickness can be increased with a relatively fewer number of LBL deposition cycles (Figure 8d), as opposed to conventional polyelectrolyte based multilayered systems. This approach is highly modular owning to the versatility of LBL as an approach, and due to use of polymer-based building blocks. Therefore, different chemical (e.g., hydrophilicity/hydrophilicity, charge interactions) or physical modifications (e.g., barrier thickness, mesh size) can be easily incorporated, to tailor networks with specific characteristics.

**Figure 9:**
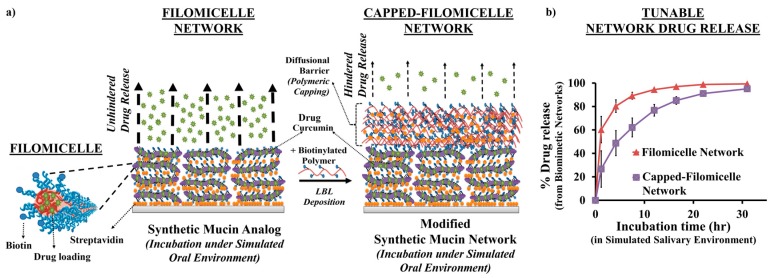
Potential synthetic mucin analogs as a tunable drug releasing network (**a**) Scheme showing use of filomicelle networks formed from crosslinking of biotinylated micelles with streptavidin, as a synthetic mucin analog. And, modification of those analog systems using polymeric capping barriers formed atop synthetic mucin. Capping barriers are formed from crosslinking of biotinylated polymer poly(acrylic acid) with streptavidin via LBL depositions; (**b**) Figure shows modification of drug release capacity from synthetic mucin systems for localized oral drug delivery applications. By developing polymeric capping barrier, the drug release from micelle-based network was greatly hindered, suggesting its capable potential to serve as tunable release systems for mucosal specific regenerative applications such as xerostomia and oral mucositis. Reprinted with permission from Reference [84]. Copyright © 2015, Wiley-VCH Verlag GmbH & Co. KGaA, Weinheim.

By utilizing PEG based diblock copolymers, these synthetic mucin scaffolds displayed excellent surface wettability (Figure 8f), with contact angles comparable to natural mucin coats. Additionally, by utilizing its porous topography, the scaffolds improved the bacterial adhesion. By incorporating anti-bacterial drug loading (e.g., curcumin) within micellar core, they can demonstrate bacterial inhibition. Since, the framework for scaffold development involve the use of drug loaded nanocarriers, the synthetic mucin networks can translate into a functional coating capable of localized drug delivery with added biomimetic interfacial characteristics (Figure 9) [84].

In a more recent study, Fiegel *et al.*, (2013) [207] developed a model of tracheal mucus. To mimic the bulk chemical and interfacial properties as the natural counterpart, the model mucus were formulated from constituents derived from tracheal mucus (glycoproteins, proteins, lipids, ions, and water). The constituents were cross-linked into a viscoelastic gel via the use of a bifunctional crosslinking agent. By controlling the crosslinker amounts, the viscoelastic properties of the synthetic mucus can be tailored to mimic variations in native mucus. Likewise, a study by Lagarce *et al.*, (2014) [208] used *in vitro* 2D and 3D models to study drug nanocarrier diffusion across mucin. In the 2D model, a mucin monolayer was formed, while the 3D model was developed through multiple sequential depositions, forming thick mucus barriers (446 μm) over Transwell^®^ membranes. The study used duodenum and jejunum mucus extracts from pig mucosal surface, in evaluating paclitaxel loaded lipid nanocapsule diffusion across mucus. Their work suggested 2D models can be potentially used for accessing transport behavior in air-water-mucus interface, while 3D model can find more relevance in studying mucin-particle diffusional transport behavior. For practical applications, the ability of those *in vitro* models to serve as an effective alternate for *in vivo* models needs to be accessed, through correlation of results from both the model systems.

Study by Burruano *et al.*, (2002) [209,210], described use of guar gum, a polysaccharide based formulation approach as a synthetic alternate of cervical mucus. In their work, guar gum was crosslinked with borate ions to recreate the viscoelastic properties of mucus. The biomimetic tendency of those synthetic gels was studied by cross-comparing its rheological properties such as spinnbarkeit and viscocity, and suggesting its close relevance to naturally occurring cervical mucus.

Wang *et al.*, (2014) [211], in their work used reversible addition-fragmentation chain transfer (RAFT) polymerization technique for synthesizing fluorophore tagged glycopolymers. In an attempt to mimic the randomly distributed glycomonomers as seen in natural mucin glycoproteins, the study used statistical tri-component copolymers. The glycopolymers were formed from the constituents glycomonomer, *N*-(2-hydroxyethyl) acrylamide (HEAA) as spacer, and *N*-(2-aminoethyl)methacrylamide (AEMA) as targets for fluorophore functionalization. Those custom-synthesized fluorescent glycopolymers contained α-galactose or β-galactose as pendant sugar moieties, and displayed lectin-mediated binding tendency [212,213] in human respiratory pathogenic bacteria (e.g., *Pseudomonas aeruginosa* and *Staphylococcus aureus)*, thereby mimicking the bacterial adherence tendency of natural mucin.

### 7.3. Significance of Mucus Mimetic Systems

The bulk functional properties of natural mucin networks (e.g., mucoadhesion, bacterial capture, barrier properties) emanate from their complex interplay of molecular interactions and structural and chemical properties. By developing a modular structural mimic, which is capable of easily tuning its network property (e.g., morphology, mesh size, thickness, chemistry), the bulk behavior in natural mucin can be easily decoupled, allowing one to identify its relative significance. In addition, under *in vitro* settings, the surface deposition of natural mucin may not provide sufficient level of control, as it is extremely difficult to recreate the varied structures mucin can adopt throughout the body (e.g., cervical mucus, salivary pellicles, *etc.*). Alternatively, synthetic analog offer a relatively simple way to incorporate changes in the network morphology and its chemistry. For instance, by adjusting the polymer chemistry (e.g., molecular weights, pendant chain chemistries) s, the networks bulk chemistry can be tailored. For example, the glycan chains are the key contributor for mucin’s lubricous nature. The extended conformation of mucin is due to the dense molecular arrangement of glycan chains, which orients the polypeptide core from globular to a more extended-conformation, forming rod-like structures [214]. These oligosaccharide residues can be incorporated onto polymeric chains, to yield a lubricous hydrophilic barrier. Studies by Levine *et al.*, (1978) [215] and Stinson *et al.*, (1982) [216], have suggested that sialic acid present in oligosaccharide chains can aid in bacterial entrapment (e.g., *Streptococcus sanguis*, *Streptococcus mutans*). These oligosaccharide residues can be incorporated for bacterial agglutination.

Conceptually, utilizing synthetic mucin analogs as a functional model, is still in its early exploratory stage. Some of the key considerations in developing synthetic mucin analogs are (1) requires a facile approach (e.g., polymeric layer-by-layer deposition (self-assembling)/polymeric gelation (bulk cross-linking)/ controlled surface growth) in forming analogs; (2) the approach should be highly modular and versatile that is capable of developing networks with tunable structural (e.g., morphology, barrier thickness, network mesh size) and chemical property (e.g., charge, hydrophobicity); (3) ability to easily elicit physicochemical modifications in component building blocks (e.g., polymeric systems); (4) the approach should be capable of recreating the formation mechanism of natural mucin networks and (5) synthetic networks should be capable of structurally and functionally mimicking bulk properties of natural networks (e.g., mucoadherence, lubricity, microbial capture/inhibition).

By developing an effective synthetic mucin system, the analogs can play a vital role, in evaluating studies that were highly reliant on natural mucins barrier and its adhesive properties. For instance, the development of drug deliverable patches (e.g., mucoadhesive buccal patches) or for studying the transport properties across mucus barrier in evaluating trans-mucosal drug delivery. The development and use of effective synthetic model lessens our overdependence on *in vivo* and *ex vivo* testing, thereby reduces the associated costs and the need for animal sacrifices. Therefore, the analog systems can serve as a prescreening process prior to conducting on a more rigorous animal model [201,217].

Alternatively, these synthetic analog systems can be also explored for potential prospect for biological applications. The biomimetic tendency, and need for porous biocompatible scaffoldings forms a key prerequisite in development of functional materials (e.g., use of porous synthetic extracellular matrix as tissue scaffolds), or developing functional coats in rendering epithelial protection and localized payload delivery from drug loaded networks [218]. For example, in the recently published work, Authimoolam *et al.* [84] with minor polymeric barrier modifications to the synthetic mucin analog, the ability to utilize these biomimic models as a tunable drug releasing structure was illustrated (Figure 8 and Figure 9).

## 8. Conclusions

Mucin networks are found as a mucosal lining along different epithelial regions within human body. These networks are often considered as a transport barrier that needs to be overcome in many drug delivery applications. However, they play a critical functional role in impacting diverse array of physiological function. With lack of mucin barrier functionality, its effects can be readily perceived (e.g., nasal/oral/eye dryness, stomach ulcers).

Mucin network’s functional behavior, as any complex polymeric gel system, is directly linked to its structural and chemical properties, and this dependency can be altered by changes in its local environmental conditions (e.g., salt, pH conditions). Due to such complexity, there is an unmet need for an effective mucin model that can easily recreate the complex structures under *in vitro* settings. By developing polymer based biomimetic synthetic analogs, and utilizing the tunable physiochemical nature of polymeric building blocks, facile control of network properties can be achieved. Such tunable networks apart from serving as a preclinical mucin model, by utilizing its biomimetic tendency, can be looked upon for its potential in various bioapplications such as developing biomaterial coatings, or in designing drug delivery systems (e.g., drug releasing scaffolds) for tissue engineering applications.
